# On the relation between economic bubbles and effort gaps between sellers and buyers: An experimental study

**DOI:** 10.1371/journal.pone.0189359

**Published:** 2017-12-11

**Authors:** Eldad Yechiam, Amitay Kauffmann, Nathaniel J. S. Ashby, Gal Zahavi

**Affiliations:** Max Wertheimer Minerva Center for Cognitive Studies, Faculty of Industrial Engineering and Management, Technion – Israel Institute of Technology, Haifa, Israel; Groupe ESC Dijon Bourgogne, FRANCE

## Abstract

Economic bubbles are an empirical puzzle because they do not readily fit the notion of an efficient market. We argue that bubbles are associated with a conflict and a gap in the allocation of effort during negotiation by sellers and buyers. We examined 21 experimental asset markets where in one condition players could buy and sell and in the other they could either buy or sell. The results indicated that when making concurrent buying and selling decisions the mean number of asks for sellers was 71% higher than the number of bids for buyers. Similar findings emerge in a re-analysis of data from Lei et al. (2001). Importantly, bubbles only emerged in markets where the number of asks was larger than that of bids. These findings indicate that bubbles are associated with increased negotiation effort when acting as a seller and diminished effort when acting as a buyer.

## Introduction

Economic bubbles are an empirical puzzle because they do not readily fit the notion of an efficient market (e.g., as formalized by [1,2]). Bubbles represent extended periods in which the price of an asset is above the fundamental (or intrinsic) value of the asset. Some argue that during the past century economic bubbles have been too frequent and large for an efficient market to make up for them, suggesting that the efficient market hypothesis should be rejected [3]. Alternatively, the ubiquity of bubbles may suggest a less restrictive interpretation of the efficient market hypothesis whereby efficiency is determined by what is known about the asset, but that relevant knowledge could be distorted or incomplete [4]. Both approaches imply that in order to understand the emergence of bubbles one should gain an understanding of the cognitive processes underlying price construction [5,6]. Asset markets with double auction design [7] have been used extensively as an experimental model to examine these processes [8,9]. We suggest that the heterogeneity between the two cognitive roles taken by traders in experimental asset markets (and most real world markets), that of a buyer or a seller, plays a central role in how market values are constructed, and contributes to the emergence of bubbles. Specifically, we suggest that a selling perspective leads to more effort than a buying perspective, as evidenced by greater number of selling proposals (i.e., asks) than buying proposals (i.e., bids), and that this gives rise to buyers being persuaded to buy at irrationally high prices.

In light of the surprising ubiquity of economic bubbles, understanding the formation and mitigation of bubbles has been the subject of extensive experimental research. Still, most studies of experimental asset markets have focused on processes shared by buyers and sellers [5–7, 10–15], such as learning from experience and forecasting future changes. By contrast, studies that have focused on cognitive differences between buyers and sellers have not examined their relation to bubbles (e.g., [16–18]).

Buyers and sellers’ effort is for all likelihood associated with their pricing performance. There is an extensive literature in psychology on the relationship between effort and performance [19–23], which is typically argued to follow an inverted U-shaped pattern (following the so called Yerkes-Dodson law [19, 24]). In the context of economic bubbles, it has been found that traders who are more cognitively reflective tend to produce lower economic bubbles [5, 6]. This may suggest that when individuals exhibit ample cognitive effort this reduces bubbles irrespectively of a person’s trading perspective. We propose, in contrast, that bubbles are a result of a conflict and a gap in cognitive effort between two psychological perspectives: Increased effort in buying generally leads to reduced prices, while increased effort in selling has the reverse effect, increasing prices. Additionally, we suggest that sellers’ effort outweighs buyers’, leading to elevated prices and bubbles.

Several empirical findings support the notion that selling is associated with greater effort. First, the literature suggests that sellers view the task of trading more from the point of view of preventing a loss (of the object) than from that of obtaining a gain, while buyers view the task more from a gain than a loss perspective [16, 25]. For example, using an implicit attitude test, a selling experience was more closely associated with losing while a buying experience was more closely associated with gaining [25]. A selling perspective may consequentially capture more attention and lead to more effort due to the effect of losses on attention (see reviews in [26, 27]). Embedding losses within a cognitive task was found to increase physiological arousal (e.g., [27, 28]), as well as search of the available alternatives [29, 30] and cognitive performance (e.g., [27, 31–33]). If selling is viewed as a preventative action then it should be conducive to similar increases in attention and performance. Also, under disequilibrium theory, individuals should prefer to engage in the behavior to which normally there is no access to [34–35]. Hence, since for most people buying experiences are relatively common whereas selling experiences are not possible, people are expected to engage more effort in selling than buying, and this is expected to sway the market to the sellers’ side.

Consistent with the notion that selling is associated with more cognitive effort, studies have shown that in pricing of lotteries participants acting as sellers tend to be more sensitive to the lottery’s expected value than those acting as buyers (e.g., [36–38]) and also tend to deliberate longer [37]. In the context of asset markets, a surplus of effort while negotiating prices may aid sellers in persuading buyers, thus elevating trading prices, and potentially leading to bubbles.

These theoretical accounts also suggest some moderators for the postulated cognitive differences between buyers and sellers. Increased attention and effort on the part of sellers should be more impactful when attentional resources are limited. This is implied by the diminishing marginal benefit of attentional investment [19,23]. Additionally, differences in attention come into play more strongly when tasks are performed concurrently, as this leads to a competition of priorities [19,20]. A case in point is where a person devotes time to both buying and selling compared to being involved in only buying or only selling. Concurrent buying and selling is therefore expected to yield greater effort gaps between buying and selling perspectives, compared to a task with only one of these activities. The same prediction is also implied by Porter and Smith’s (1994 [39]) Active Participation Hypothesis, which suggests that when only one pricing activity is possible (i.e., either buying or selling) individuals usually put their effort into it (see also [40]).

In the current study we evaluated these predictions in an experimental asset market. In order to study the effect of concurrent buying and selling we compared a market where traders either buy or sell versus one where they can perform both tasks. As a proxy for negotiation efforts, we examined the volume of price proposals (i.e., bids and asks) which is considered to reflect the extent of one’s efforts at negotiating different prices [39,40]. Consistent with this notion, the volume of price proposals was shown to decrease when participants reduced their effort due to a secondary task of participating in a second market in parallel [40]. In line with the proposed effort gap between buyers and sellers, we expected that in double auction experimental markets the mean number of asks would be larger than the mean number of bids. A larger number of asks than bids was previously observed in a double auction market study where agents could simultaneously take short and long positions [41]. Additionally, we predicted that having a larger number of asks than bids would be associated with higher proposed and accepted prices for both buyers and sellers, and ultimately with market prices in excess of the fundamental value of the asset. Consistent with this notion, in studies of call markets making the first proposal was found to be associated with greater negotiation success [42–44]. Finally, we examined the volume of accepting others’ price proposals (i.e., the degree of responsiveness) sometimes referred to as “market order”. We expected that due to sellers’ greater effort, buyers would be more likely to accept sellers’ proposals than vice versa.

## Reanalysis of data

**Fig 1:**
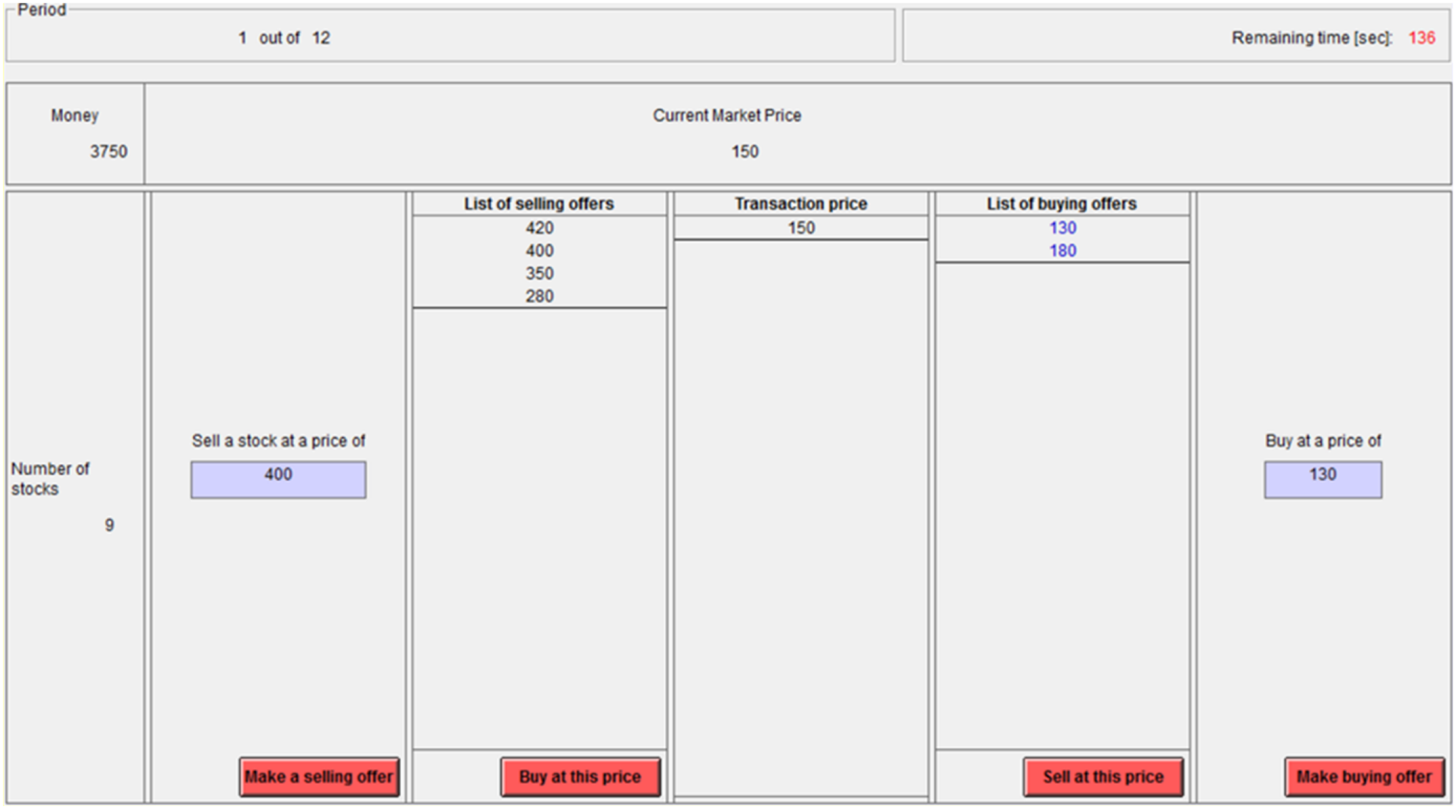
An illustration of the asset market task. The top panel indicates the current round and remaining time (in seconds). The middle panel shows the current amount of money owned and the market price (price of last transaction). The bottom panels indicate (left to right) the number of owned assets (9), an ask (a selling proposal for 400 tokens), a list of all available asks, a list of all transaction prices (ordered by time), a list of all available bids, and a bid (a buying proposal for 130 tokens). The buttons at the bottom (left to right) are for making a selling proposal, accepting a selling proposal, accepting a buying proposal, and making a buying proposal.

**Fig 2:**
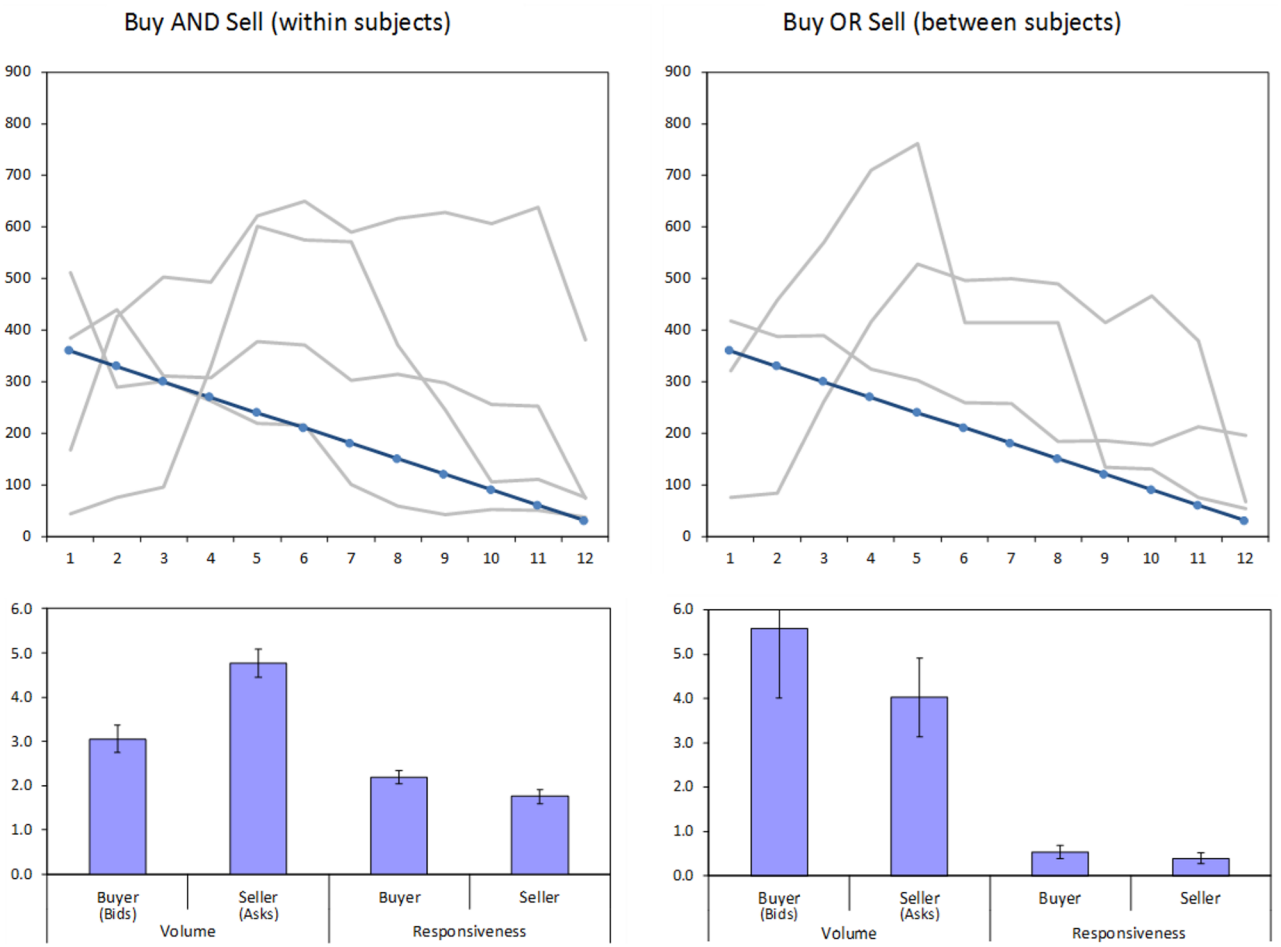
Re-analysis of Lei et al. (2001 [40]). Top panels: Average market prices in 12 rounds of trade, in the Buy AND Sell and Buy OR Sell conditions. The gray lines represent different markets (sessions) and the blue line the fundamental value of the asset. Bottom panels: Volume of price proposals (number of bids and asks per round) and responsiveness (number of acceptances of others’ proposals per round) for buyers and sellers in each condition. Error terms denote standard errors.

To pilot these research questions, we re-analyzed data from a standard experimental asset market [7] collected by Lei et al. (2001 [40]). The re-analysis focused on the experiments in Lei et al. (2001 [40]) involving a single asset market. Lei et al. (2001 [40]) examined two conditions, one where participants could both buy and sell assets (Buy AND Sell), and another where some participants could only buy while others could only sell (Buy OR Sell). The second condition was added in order to eliminate the possibility that bubbles emerge due to mere speculation (buying the asset for the purpose of selling it later at a higher price). The experiment was administered to 51 individuals (in seven groups of 7–8 participants). The task–illustrated in Fig 1—included 12 trading rounds using continuous double auction rules [7; and see Methods section), with each round lasting 4 minutes. Lei et al. (2001 [40]) found considerable bubbles, namely market prices above the fundamental value (FV) of the asset, in both conditions (see top panels in Fig 2).

Using Lei et al.’s [40] data, we first examined whether there is an asymmetry in the volume of price proposals, namely in the mean number of asks compared to bids (see the bottom panels of Fig 2). In the Buy AND Sell condition there was a significant difference between these indices, with the mean number of asks being 56% larger than the mean number of bids (*t*(27) = 2.75, *p* = .01). There was no significant difference between the number of asks and bids in the Buy OR Sell condition (*t*(21) = 0.65, *p* = .52).

We then compared markets where the mean number of asks was larger than that of bids, to those where the number of bids was larger (there were five markets in the former and two in the latter category). Buying price proposals in markets dominated by asks were somewhat higher compared to their counterparts, though the effect was only marginally significant (mean proposed prices of 223.09±20.63 vs. 164.99±21.81, respectively; *t*(36) = 1.94, *p* = .07). High buying proposals significantly predicted excessive transaction prices above the fundamental value (price—FV; *r*(36) = 0.38, *p* = .02); however, there was no direct relation between the market’s asks minus bids index and excessive transaction prices (*r*(48) = 0.15, *p* = .30). The latter analyses are sensitive to confounds since they are pooled across the two experimental conditions (Buy AND Sell vs. Buy OR Sell). However, the sample size was too small to conduct an analysis controlling for condition (n’s ≤ 10 in some of the cells). In our main study we conducted a more thorough analysis that controls for experimental condition.

Though based on a small sample of participants, these re-analyzed prior findings indicate a considerable disparity in the volume of price proposals put forth by buyers and sellers, with the number of asks being larger than the number of bids. A second observation is that this disparity was only significant when participants both bought and sold concurrently. Additionally, higher numbers of asks compared to bids predicted the size of buying proposals. This pattern is consistent with the theoretical accounts detailed above suggesting that selling activities are prioritized in conditions involving concurrent buying and selling, in which there is competition between priorities and limited cognitive resources. In the current experiment we sought to examine whether the disparity between asks and bids emerges in a considerably larger sample and to clarify its relation to market bubbles.

## Methods

The data was gathered in twenty-one experimental sessions conducted at an Israeli University. In total, there were 165 participants, 103 men and 62 women. All participants were students, recruited via an online system, posters, and announcements. Very few participants had any trading experience (13%) and none had prior experience in asset market experiments. Their average age was 24.7; ages ranged between 18 and 40. Experimental sessions lasted approximately 75 minutes, and payment ranged between NIS 25 and 100 depending on performance. There were eight participants in each session; three sessions included only seven participants each due to insufficient show ups.

Following Lei et al. (2001 [40]) participants were randomly assigned to one of two conditions: “Buy AND Sell” or “Buy OR Sell”. Because there is less data in the latter condition, the number of sessions was preset so that there were 9 sessions in the Buy AND Sell condition and 12 in the Buy OR Sell condition. Within the Buy OR Sell condition participants were randomly allocated to the role of buyer or seller (48 buyers and 47 sellers).

The experimental asset market was based on the parameters of Lei et al.’s (2001 [40]) experiment. The experimental asset had a finite lifespan of 12 rounds (periods). At the end of each round, each asset paid a dividend that was randomly determined to be worth either 20 or 40 tokens. Hence, the expected future dividend at round *t* equaled the expected value of the dividend (30 tokens) multiplied by the remaining rounds (30 × (13-*t*)). The number of assets in one’s possession and the cash balance were accumulated during rounds. The asset had no value for the participants after the experiment had ended, while experimental currency was converted into real currency at a rate of NIS 1 per 200 tokens.

In the Buy OR Sell condition participants were assigned to the role of either buyers or sellers. Buyers were only permitted to purchase, while sellers were only permitted to sell. At the beginning of the experiment, each seller was endowed with 20 units of the asset (referred to as “stocks”) but no experimental tokens. Each buyer was endowed with 7,200 tokens (referred to as “Talers”) and no units of the asset. Because each unit of the asset paid on average 360 tokens during the 12 rounds, the expected value of the initial endowment was identical for buyers and sellers in this condition. In the Buy AND Sell condition all participants could buy and sell, and each participant was endowed with 10 units of the asset and 3,600 tokens at the beginning of the experiment. The expected value of the initial endowment in this condition was thus the same as in the Buy OR Sell condition.

The sequence of events in all sessions was as follows. Upon arrival, participants completed an informed consent statement and received written instructions consisting of a description of the task and an explanation of the dividend structure. The instructions were based on those described in Lei et al. (2001 [40]), translated into Hebrew. Briefly, the instructions explained the basic actions in the experiment (buying, selling, accepting another’s buying and selling proposals), how dividends were allocated, the conversion rate, the number of trading periods, and the number of participants in the current session. Participants were asked to avoid making price proposals when they did not have a sufficient number of assets (i.e., short sell), and to maintain a positive cash balance. If during the experiment a price proposal was inconsistent with these constraints, the experimental program issued a message explaining that this was an erroneous proposal. Participants were further asked not to make subsequent trailing price proposals with the same exact value before a trade was made on a previous proposal (as in [40]). This request was made in order to restrict a strategy of entering the same price proposal over again (e.g., in order to increase the visual load and confuse other players). Still, as the proposals were ordered according to their favorability such a strategy was expected to have limited effects.

The experimenter read these instructions out loud. Participants were then encouraged to ask questions. This was followed by a short quiz regarding the value of a single asset one round ahead and 12 rounds ahead. If participants did not complete the test properly they were given further explanation until they were able to answer correctly. Next, there was a 4 minute training session in which participants practiced using their designated perspective in the experimental task (i.e., as either sellers, buyers, or both). The initial endowment in the training session was identical to that given in the main experiment but monetary decisions were not incentivized. Participants then received a piece of paper stating their initial endowments and the possible actions available to them (either buying or selling in the Buy OR Sell condition, or both in the Buy AND Sell condition). This was followed by the 12 rounds asset market experiment with continuous double-auction rules.

Fig 1 includes a screenshot from the experimental program, designed using z-Tree [45]. As illustrated in Fig 1, participants could create multiple bids and asks in each round that were visible to all players in their session, and could also accept pending proposals created by other players. Each round lasted 4 minutes. The bids and asks boxes could include up to 44 price proposals each, sorted such that the most attractive ones (highest bids, lowest asks) were at the bottom while the least attractive ones were at the top. Participants could select the most attractive bids and asks (those at the bottom of the list), or not. If more than 44 price proposals of a given type were made, the initial box was turned into a scrollable box with room for an additional 44 proposals that could be observed by scrolling up. Because the box automatically scrolled down, the most attractive price proposals were observable to all participants by default. This was designed to prevent a strategy of making multiple unattractive proposals so as to eliminate the presentation of attractive proposals (as in [7]).

Once a bid or an ask of a given participant was accepted by another all relevant pending price proposals of these participants were removed. For example, if a participant *A* accepted a pending bid from participant *B* then all asks for *A* and all bids for *B* were removed. Thus, each price proposal pertained to a single unit of the asset (as in [7]). At the end of the experiment, assets were nullified and participants received money according to the number of experimental tokens in their possession (200 tokens = NIS 1).

As in our re-analysis of Lei et al. (2001 [40]), we calculated the mean number of bids and asks, and of acceptances of others’ bids and asks in each round. In line with the experimental instructions and the procedures in Lei et al. (2001 [40]) this did not include trailing proposals in which the same prices were repeated consecutively before an asset was purchased. To evaluate pricing decisions, we examined mean proposed prices and mean accepted prices by buyers and sellers in each round. To evaluate market bubbles, we examined differences between the mean price of transactions and the fundamental value of the asset (which changed each round). This is a standard index that can be applied at the participant level, allowing for increased statistical power and examination of individual differences [13,15,40]. Additionally, in order to gain better insight into the role of different perspectives on excessive pricing we further divided this index into transactions that were accepted by sellers and buyers based on the other party’s proposals.

For descriptive purposes, we also calculated two common market-level indices of bubbles [13, 40]: Bubble duration, indicating the number of rounds where the market price (mean transaction price) was above the fundamental value; and bubble amplitude, which captures the disparity between the maximal and minimal departures from the fundamental value, as follows:

$$\text{Amplitude}=\underset{t}{\text{max}}\left(\frac{\text{Pr}ice_{t}-FV_{t}}{FV_{t}}\right)-\underset{t}{\text{min}}\left(\frac{\text{Pr}ice_{t}-FV_{t}}{FV_{t}}\right),$$ (1)

Where Price(*t*) refers to the mean transaction price in round *t*, and FV(*t*) refers to the fundamental value in round *t*.

## Results

### Market level price patterns

**Fig 3:**
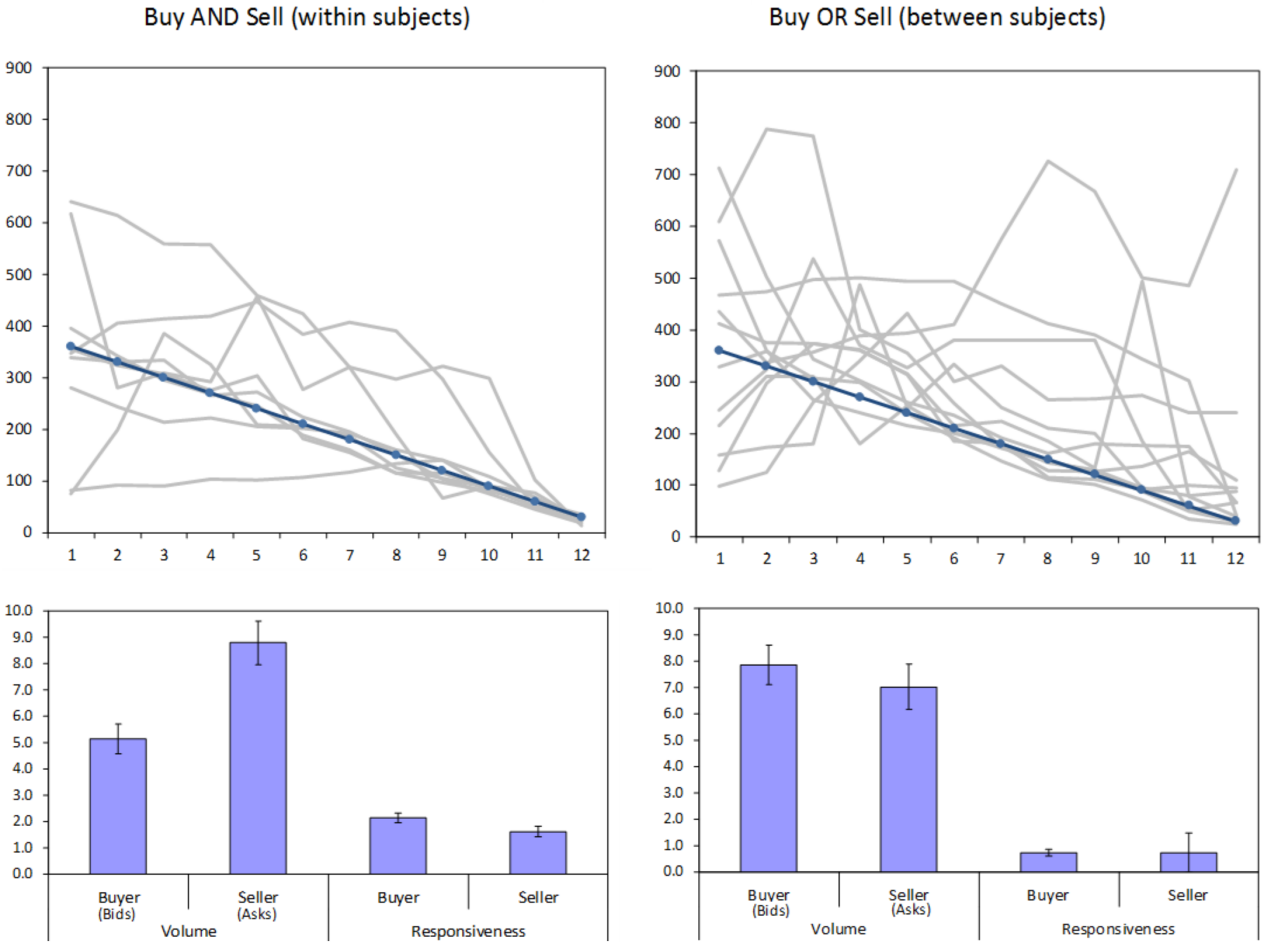
Study results. Top panels: Average market prices in 12 rounds of trade, in the Buy AND Sell and Buy OR Sell conditions. The gray lines represent different markets (sessions) and the blue line the fundamental value of the asset. Bottom panels: Volume of price proposals (number of bids and asks per round) and responsiveness (number of acceptances of others’ proposals per round) for buyers and sellers in each condition. Error terms denote standard errors.

The mean prices in each trading round appear in Fig 3. As can be seen, there was large variance between sessions but overall prices tended to be somewhat higher than the fundamental value (FV) of the asset. In all, there were 157 rounds in which the asset was traded above the FV, compared to 95 rounds in which it was traded below the FV. The mean value that the asset was traded at across rounds was 251.3±20.21 tokens compared to an FV of 195 tokens (*t*(21) = 2.79, *p* = .01). The results are thus consistent with those of Smith et al. (1998 [7]) and Lei et al. (2001 [40]) in that significant bubbles emerged. On average, the maximal duration of bubbles in a given session was 6.3±0.81 rounds, which indicates that the largest continuous bubble in a given session spanned about half of the experimental rounds. The mean bubble amplitude was 2.98±1.06, implying that differences between maximal and minimal price deviations from the FV ranged on average about three times the expected value of the asset.

We also examined whether differences in the market indices of bubbles emerged between conditions. For both indices of bubble duration and bubble amplitude there was a marginally significant difference between conditions, with longer and more varied bubbles in the Buy OR Sell condition than in the Buy AND Sell condition (Duration: *t*(19) = 1.82, *p* = .084; Amplitude: *t*(19) = 1.84, *p* = .091).

Another difference between market conditions was the number of transactions in each condition, which was considerably higher in the Buy AND Sell condition (3.78±0.36) than in the Buy OR Sell condition (0.72±0.07). This difference was significant even at the market level (*t*(21) = 8.14, *p* < .001).

### Effect of perspective on negotiation indices

As shown in Fig 3, the differences between bids and asks replicated those found in our re-analysis of Lei et al. (2001 [40]). In the Buy AND Sell condition the mean number of asks was 71% higher than the number of bids (paired-sample *t*(69) = 4.80, *p* < .001). There was also a significant difference in responsiveness in the predicted direction, with buyers being 32% more likely to accept an ask than sellers were to accept a bid (paired-sample *t*(69) = 2.81, *p* = .006). As in the data of Lei et al. (2001 [40]), these trends were not found in the Buy OR Sell condition, where no significant differences emerged between buyers’ and sellers’ bids and asks (*t*(93) = 0.74, *p* = .46) and their responsiveness (*t*(93) = .07, *p* = 0.94).

The effects in the Buy AND Sell condition did not seem to be due to extreme values. The median number of asks in the Buy AND Sell condition was 6.71 compared to 3.58 for bids, and the main effect was replicated in a non-parametric test (Signed Rank *W* = 4.92, *p* < .001). Similarly, the median buyer responsiveness was 1.65 compared to 0.92 for sellers, a significant difference (Signed Rank *W* = 2.94, *p* = .003). An analysis of changes in the mean number of bids and asks over time (in different rounds) appears in the S1 Supplementary section.

### Relation between negotiation indices and bubbles

**Fig 4:**
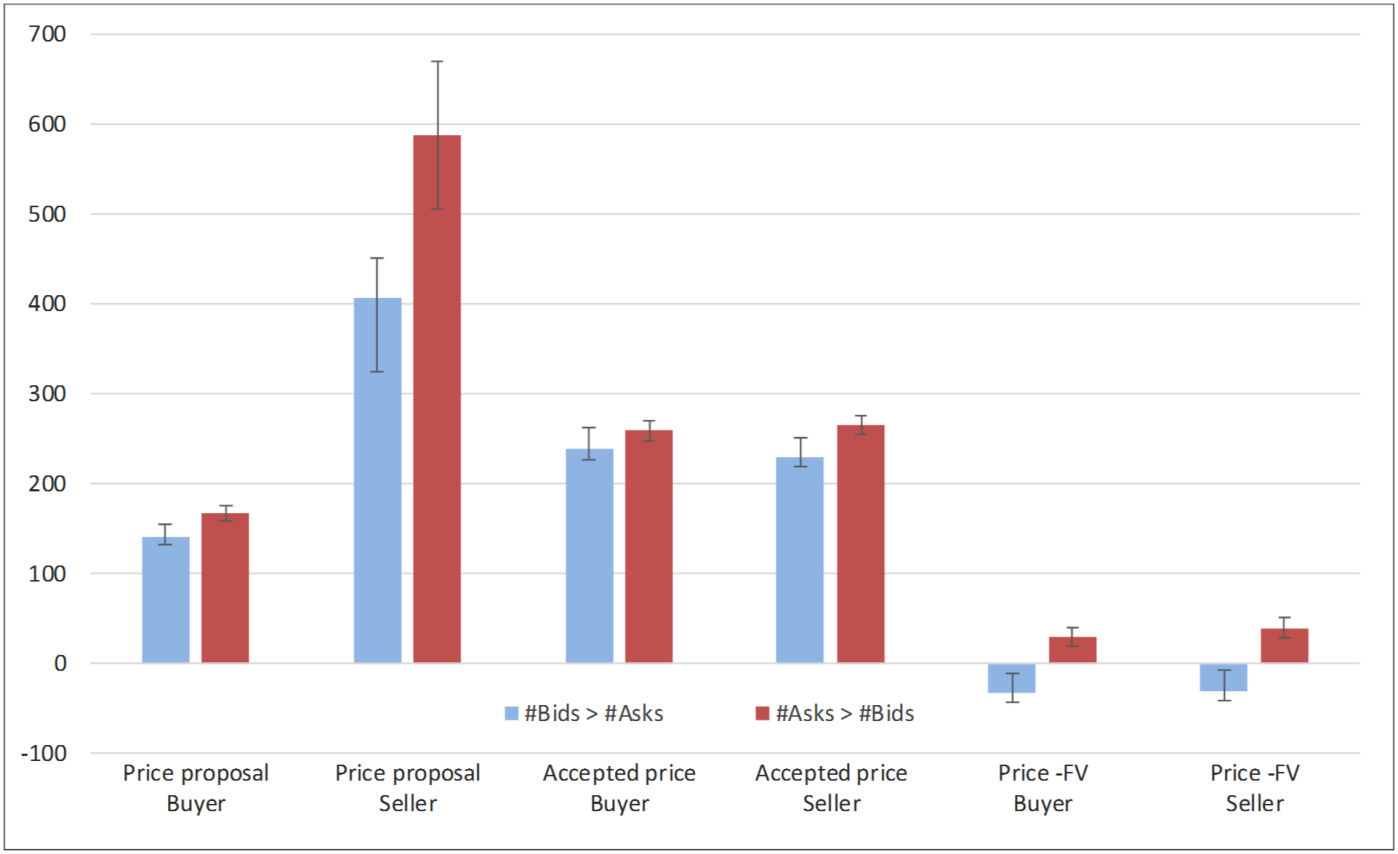
Differences between markets with higher numbers of bids or asks in mean proposed prices, accepted prices, and transaction prices in excess of the fundamental value (Price—FV). Buyers and sellers’ pricing decisions are shown separately. Error terms denote standard errors.

Next we examined the pricing implications of the asymmetry between the number of bids and asks. For this purpose, we compared markets (sessions) characterized by more asks than bids, versus those with more bids than asks. This enabled a direct contrast of an environment where selling or buying proposals are more dominant. In all, there were 13 markets where asks outnumbered bids and 8 markets where bids outnumbered asks (there were no markets with equal numbers of bids and asks). The mean pricing characteristics of the two groups are shown in Fig 4. Respective differences in these indices were tested by means of General Linear Model analyses, with market type with respect to number of bids to asks (1 = #Asks > #Bids, 0 = #Bids > #Asks) and condition (Buy AND Sell and Buy OR Sell) as independent factors.

As in the data of Lei et al. (2001 [40]), markets with higher number of asks than bids were characterized by higher buying proposals (*F*(1,113) = 6.07, *p* = 0.01) and also by higher selling proposals (*F*(1,113) = 4.04, *p* = 0.047). Buyers in these markets also accepted higher price proposals (*F*(1, 98) = 6.34, *p* = .01), as did sellers (*F*(1, 97) = 6.49, *p* = .01).

Markets with higher number of asks were further characterized by higher positive deviations from the FV (price—FV), both when transactions were accepted by buyers (*F*(1, 112) = 11.26, *p* = .001), as well as by sellers (*F*(1, 113) = 16.65, *p* < .001). Indeed, for markets dominated by higher number of asks transaction prices were significantly above the FV (transactions accepted by buyers: *t*(81) = 2.72, *p* = 0.008; by sellers: *t*(81) = 3.53, *p* = 0.010). In contrast, in markets dominated by higher numbers of bids transaction prices accepted by either buyers or sellers were not significantly different from the FV (*t*(33) = -1.50, *p* = 0.14; *t*(33) = -1.36, *p* = 0.18, respectively). Thus, transaction prices significantly exceeded the FV only in those sessions that had greater numbers of asks than bids. As shown in Fig 4 (rightmost bars), the positive difference between the mean transaction price and the FV in markets dominated by asks was about equal to the negative difference in markets dominated by bids, but the latter was more noisy. At the aggregate level, however, because there were more sessions with higher numbers of asks, overall prices tended to be above the FV.

Finally, in line with the differences observed at the market level for bubble duration and amplitude, there was also an effect of condition on excessive prices for transactions accepted by sellers: The positive disparity between transaction prices and the FV was higher in the Buy Or Sell condition than in the Buy AND Sell condition (*F*(1,113) = 6.51, *p* = .01). No such effect emerged for transactions accepted by buyers (*F*(1,113) = 2.68, *p* = .11). We also looked at the mean price of the transactions at the market level in markets with more asks than bids and vice versa. FV. When regressing market price on condition and market type, there was a positive association between asks-dominated markets and mean market price (*r* = 0.33, *p* = 0.20). The effect was not significant, however, likely due to poor statistical power (*df* = 19).

There was no effect of condition on the magnitude of buying and selling proposals (*p* = 0.1, 0.13, respectively). However, the Buy OR Sell condition was associated with higher accepted prices both for buyers and for sellers (*F*(1, 98) = 15.39, *p* < .001; *F*(1,97) = 4.46, *p* = .04). With respect to the magnitude of buying proposals, there was also an interaction between condition and selling to buying volume (*F*(1,113) = 9.57, *p* = .002) with the effect of market type being stronger in the Buy AND Sell condition. A similar interaction emerged for the price accepted as a seller (*F*(1,97) = 7.00, *p* = .01) and for excessive prices above the FV in transactions accepted by buyers (*F*(1,97) = 5.26, *p* = .02).

### Relation of negotiation indices to individuals’ pricing decisions

The Buy AND Sell condition allows assessment of the relation between individuals’ pricing decisions and the volume of their bids and asks. We therefore examined whether individuals’ pricing decisions were predicted by the disparity between their own number of asks compared to bids (#Asks–#Bids). The results indicated that the difference between the number of asks and bids was strongly correlated with the magnitude of buying proposals for a given individual (*r*(67) = 0.40, *p* < .001) and showed a marginally significant correlation with the average price accepted as a buyer (*r*(63) = 0.21, *p* = 0.08). The difference between the number of asks and bids was also marginally correlated with excessive prices above the FV (price—FV) in transactions accepted by buyers (*r*(67) = 0.21, *p* = .08), but not sellers (*r*(68) = 0.05, *p* = 0.68).

Given these individual differences it was interesting to evaluate to what extent the tendency of transaction prices to exceed the FV in the Buy AND Sell condition was predicted by the individual’s index of #Asks–#Bids, or by the markets’ (session’s). To examine this, we conducted a regression analysis for predicting excessive transaction prices (price—FV) using two variables: 1) whether a session had higher number of asks or bids (calculated as in the section above), 2) the individual’s #Asks–#Bids. The results are presented in Table 1. As shown in the table, the market indicator with respect to having more asks than bids was the only significant predictor of excessive transaction prices, in transactions accepted by both buyers and sellers. Thus, it appears that there was no direct relation between individuals’ asks to bids difference and excessive transaction prices. Partly, though, individuals’ volume of price proposals predicted their session volume (*r*(163) = 0.25, *p* = 0.001).

**Table 1:** Results of regression analyses for the Buy AND Sell condition.

	Excessive price (Price—FV)	
	Accepted by buyers	Accepted by sellers
(constant)	-253.83	-290.64
Asks to bids—Market+	140.03 (34.90)**	167.04 (39.08)**
Asks to bids—Individual	1.81 (1.66)	-9.51 (26.82)
Model adjusted *r*^2^	0.21**	0.22**

The two columns denoted the predicted variables: Excessive transaction price (Price—FV) for transactions accepted by buyers and sellers. The rows indicate the two predictors: Disparity between the number of bids and asks at the market and individual level. Entries represent estimated regression coefficients.

^+^ = For market differences: 1 = #Asks > #Bids, 0 = #Bids > #Asks; for individual differences: #Asks—#Bids.

** = *p* < .001

## Discussion

The current findings indicate a basic difference between the volume of buying and selling proposals: When individuals can act as both sellers and buyers, the number of asks far exceeds the number of bids. Secondly, in our new study the disparity between the number of asks and bids appeared to predict the magnitude of bubbles. In markets dominated by asks, buyers as well as sellers had higher price proposals and accepted higher prices, and extant bubbles developed, as evidenced in transaction prices being above the fundamental value of the asset. In markets dominated by bids, there were no significant differences between transaction prices and the fundamental value of the asset. These findings echo those observed in our re-analysis of Lei et al.’s (2001 [40]) experiment, and are consistent with the view that bubbles are driven by a conflict between the two trading perspectives, and the tendency of the selling perspective to be more dominant and attract more attention.

Differences in the numbers of bids and asks did not emerge when participants could only buy or only sell. This is consistent with notion that a selling perspective captures more attention when there is a competition in priorities owing to the limited cognitive resources available [23]. Still, an alternative explanation for the difference between conditions is that in the sessions where individuals could both buy and sell there was a self-selection process such that those traders who chose to engage in more effort (or were more sophisticated) made more price proposals and transactions in the seller side than in the buyer side. By contrast, the same did not occur when individuals were randomly placed in a condition where they could either buy or sell.

Interestingly, despite the fact that larger differences between numbers of bids and asks emerged in the Buy AND Sell condition than in the Buy OR Sell condition, excessive transaction prices were higher in the Buy OR Sell condition (for transactions accepted by buyers). This indicates that other factors besides the volume of proposals modulate the difference between conditions with respect to excessive pricing. One relevant factor may be trading experience. Because assets in Buy AND Sell condition could be rebought and resold, participants gained much more trading experience in this condition (as reflected in the larger number of transactions). It is well known that experience moderates the emergence of bubbles [8,13,46]. For example, Lei and Vesely (2009 [46]) found that when participants observed and earned dividends from an asset in a pre-market phase this reduced bubbles in subsequent trading. Another possible reason for the increased bubbles in the Buy OR Sell condition may be the need of buyers to actively participate in the market: In this condition they have no choice but to do so through buying assets, though their price may be inflated [40].

The present findings constitute a departure from the standard assumptions about buying and selling proposals in economic theory. The standard way to interpret price proposals in economics is that a surplus of asks represents high supply, leading to reduced prices; whereas a surplus of bids represents high demand, leading to increased prices [47,48]. We find the opposite pattern: The number of asks predicts increased market prices and the emergence of bubbles. This finding can be explained based on the notion that price proposals are not only indicators of the scarcity of the product, but are also used as salient cues for all participants in the market. Due to processes such as anchoring and adjustment and social comparison, one’s price proposals affect others’ pricing decisions [49,50].

One clear implication based on the current findings therefore concerns the relation between price proposals and bubbles. The findings suggest that reducing the gap between the numbers of bids and asks might be helpful in reducing bubbles. Obviously, in actual asset markets involving non-algorithmic trading, it is not possible to affect differences in the number of bids and asks without regulating the market. However, one could potentially reduce asymmetries by modifying the salience of bids and asks, for instance by using distributed averaging algorithms or a closed order book wherein only the current bid/ask spread is presented. Examining the effect of such presentation changes on asymmetries in pricing is an interesting endeavor for future studies.

An obvious limitation of our research is that it focuses on experimental asset markets. First, in this setting participants have relatively little experience: With extended experience, differences between buyers and sellers, such as the endowment effect, tend to diminish [51–53]. Second, the experimental asset market includes unordinary assets and this is also likely to contribute to the emergence of gaps between buyers and sellers [54]. The generality of the current findings to naturally occurring asset markets (including ones involving tangible consumer products) should therefore be evaluated. A further limitation is that we have only studied a monotonically decreasing fundamental value path. Examinations of different fundamental value paths would yield a more detailed appraisal of the relationship between bid-ask imbalance and mispricing. A final limitation concerns the endogenous assignment of markets into those dominated by asks or bids. Owing to this assignment, while we have demonstrated that the asymmetry in volume of proposals predicts the emergence of bubbles, the reverse pattern whereby mispricing causes the asymmetry in price proposals is also plausible. Additional experiments should be conducted to verify the direction of causality.

In sum, in the studied experimental asset markets, the outcome of one’s effort in negotiating prices through price proposals depended on one’s trading perspective. Greater numbers of asks than bids were predictive of the emergence of bubbles, whereas greater numbers of bids than asks were not. More markets conformed to the former type, however, giving way to bubbles on the aggregate level. The findings highlight the relevance of theories of attention and effort to studies of economic markets.

## Supporting information

S1 Text Differences between bids and asks over time. (DOCX)
