# Supplementary material for: On the relation between economic bubbles and effort gaps between sellers and buyers: An experimental study
Source: PLoS One. 2017 Dec 11;12(12):e0189359. doi: 10.1371/journal.pone.0189359 (PMC5724868; doi:10.1371/journal.pone.0189359)
Supplement: S1 Text — (DOCX) [file pone.0189359.s001.docx]

**S1: Differences between bids and asks over time**

In addition to studying differences between the number of bids and asks across rounds, we tested whether these differences decrease with experience in the Buy AND Sell condition. As shown in Figure A1, the gap between the number of bids and asks tended to be stable across rounds, while differences in responsiveness between sellers and buyers abated somewhat with experience. An all-within General Linear Model with perspective (buyer, seller) and round as predictors was conducted to examine whether in addition to the effect of perspective on these indices, there is an interaction between round and perspective. The results indicated a significant effect of perspective on the number of bids and asks (*F*(1,67) = 23.03, *p* < .001), but showed no effect of round (*F*(11, 737) = 1.06, *p* = .41), or interaction between perspective and round (*F*(11, 737) = 1.34, *p* = .23). For responsiveness, the analysis indicated an effect of perspective (*F*(1,67) = 7.90, *p* =.006), with buyers tending to accept sellers’ proposals at a greater rate. There was also a main effect of round (*F*(1,67) = 3.75, *p* < .001) denoting a decreasing tendency to accept proposals with experience. However, the interaction between round and perspective was not significant (*F*(1,67) = 1.47, *p* = .17). Overall, it appears that differences between buyers and sellers with respect to the volume of price proposals (bids vs. asks) and responsiveness were consistent across trading rounds.

**Figure A1.** Round by round results for the Buy AND Sell condition. Mean volume of proposals (number of bids and asks per round) and responsiveness (number of acceptances of others’ proposals per round) for sellers and buyers.
